# Interdisciplinary rehabilitation after whiplash injury: An observational prospective 5 years outcome study

**DOI:** 10.1097/MD.0000000000006113

**Published:** 2017-03-03

**Authors:** Philipp Haiduk, Thomas Benz, Susanne Lehmann, Francoise Gysi-Klaus, André Aeschlimann, Beat A. Michel, Felix Angst

**Affiliations:** a Research Department, RehaClinic Zurzach, Bad Zurzach; b Clinic of Rheumatology, University Hospital Zurich, Zurich, Switzerland.

**Keywords:** long-term, rehabilitation, WAD, Whiplash injury associated disorder, working capacity

## Abstract

Whiplash injury associated disorders (WAD) cause high costs for public health care. Neck pain is number 16 on the global prevalence lists for the 50 most common sequelae. It is of importance to obtain long-term data on disability and working capacity outcomes after rehabilitation. Long-term prospective data of the outcome course of whiplash are sparse. The aim of this study was to quantify improvements of pain, function/role performance, vitality, and working capacity 5 years after whiplash injury and to compare the state of health to normative values at 5 years after rehabilitation.

In this naturalistic, observational, prospective cohort study, 115 patients were assessed 5 years (60 months) after a multidisciplinary rehabilitation program. The assessment set consisted of the Short Form 36 (SF-36), parts of the North American Spine Society's cervical spine assessment questionnaire (NASS) and the coping strategies questionnaire (CSQ). The effects were quantified by effect size (ES) and standardized response mean (SRM). Score differences over the course were tested by the Wilcoxon–Mann–Whitney *U* test for significance.

Comparing data between entry and 60 months after rehabilitation 8 of 15 parameters improved with large ES/SRM. Outcome between 6 and 60 months showed small to moderate ES/SRM. Working capacity increased from 0 at entry to rehabilitation to 21 h/wk at 6 months and to 30 h/wk at 60 months follow-up.

After large improvements in health and working capacity in the mid-term, further important improvements were observed in the long-term course. It can be hypothesized that part of those can be attributed to the interventions during inpatient rehabilitation, for example, due to better coping strategies.

## 1 Introduction

Whiplash injury associated disorders (WAD) cause high costs for public healthcare and can have dramatic consequences for the individual, especially in the long-term. Neck pain is listed as number 16 for the global prevalence of the 50 most common sequelae. The number of years lived with disease had increased within 20 years from 24 to 34 billion by 2010 according to the Global Burden of Disease Study.^[1]^ The incidence of persistent neck pain, for example, has been reported as 84% to 90% in 1 to 2 years and 55% in 17 years after injury.^[2]^ Return to work rate has been reported as 34% in 1 year and 43% in 3 years after WAD compared with 51% and 59% for other musculoskeletal diseases (n = 104).^[3]^ Therefore, it is important to obtain long-term data on outcome and working capacity after interventions.

Multidisciplinary rehabilitation programs have proven beneficial for patients suffering from chronic WAD.^[4–8]^ Most of the quantitative outcome studies in WAD are observational^[4–6,8]^ but randomized controlled trials (RCTs) are rare.^[7,9]^ While 1 RCT showed that exercise combined with advice is slightly more effective than advice alone, the other RCT concluded that a comprehensive exercise program, consisting of 20 sessions of exercise, was not more effective than advice alone.^[7,9]^ Our observational studies in the past examined a rehabilitation program, which is much more comprehensive than the interventions of those 2 RCTs.^[7,9,10]^ Patients improved in important health dimensions on a moderate to large scale as measured after discharge (effect sizes [ES] up to 0.87) and both short and midterm up to 6 months (ES up to 0.87) after rehabilitation.^[10]^ There are only few studies covering the outcome of participants of rehabilitation programs for WAD in the long-term.

Seventeen months after a 4-week inpatient rehabilitation program in Sweden Söderlund quantified cross-sectional outcomes based on the sickness impact profile of WAD patients, but did not analyze effects to baseline.^[11]^ In another follow-up study 12 months after an interdisciplinary outpatient rehabilitation program for WAD patients pain improved by ES 0.29 and function by 0.49 on the multidimensional pain inventory. Fulltime and part-time working capacity increased comparing baseline to follow-up.^[12]^ At the 3 years follow-up after WAD caused by motor vehicle collision a RCT calculated a standardized mean difference of 0.42 (*P* = 0.158) for the late active intervention compared with the late standard intervention. Active intervention consisted of repeated cervical rotation and other exercises, whereas standard intervention only comprised of advice on suitable activities.^[13]^ While Söderlund's study examined 104 patients all other studies mentioned had small sample sizes.

The first aim of this study was to quantify improvements in the areas of pain, function/role performance, vitality, and working capacity at 5 years (60 months) and to compare the state of health to normative values at 5 years. The second aim was to quantify changes of health between 6 and 60 months.

## 2 Methods

### 2.1 Sample

**Figure 1:**
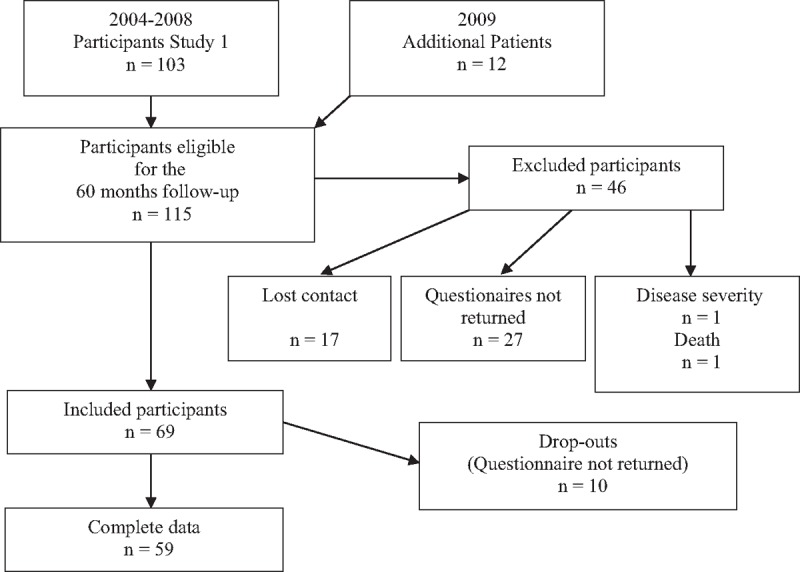
Patient selection.

This naturalistic, observational, prospective, cohort study was based on the participants of the previous mid-term cohort study.^[10]^ The patients of the previous study (n = 103) were supplemented by a further 12 patients who had all complete data between baseline and the 6 months follow-up (n = 115). All were contacted by telephone and asked to complete the 60 months follow-up (Fig. 1). In the following time, no more cases were added since a new study (with altered questionnaires) was planned. All patients referred to the rehabilitation program were asked to take part in the study. Further details of patient selection were described in the previous report.^[10]^

Inclusion criteria were: 1. Chronic neck pain of minimum 3 months and maximum 5 years duration caused by whiplash injury. 2. First time accident causing whiplash injury (re-injuries due to accident and/or others were excluded). 3. History of failed or insufficient efficiency of outpatient treatment. 4. No previous inpatient treatment for WAD in the past. 5. Age between 17 and 65 at entry. 6. Willingness to participate in the program. 7. Sufficient German language skills and cognitive abilities to understand the content and to fill out the questionnaires.

Exclusion criteria were: severe somatic illness requiring specific treatment (i.e., cancer, inflammatory rheumatic disease, spinal fracture, neurological disease, and pain after recent operation) or manifest severe psychiatric disorder. Severe was defined that the patient was not able to participate in the program.

Written signed informed consent was obtained from all participants. The study protocol was approved by the local ethic commission (Health Department Aargau, Switzerland, EK AG 2008/026).

The 4 interdisciplinary pain program focused on the cervical spine and consisted of active physiotherapy (individual and in groups: 5.5 h/wk), strength and endurance training (1–2 h/wk), occupational therapy (5 h/wk); psychology: cognitive behavioral and coping therapy (individual and in groups, 5–6 h/wk), relaxation (3 h/wk), other: music and painting therapy (2–3 h/wk), Tai Chi and Qigong (3 h/wk). All patients rested 4 weeks in the program and received all therapy elements. The program has been described in detail in a previous publication.^[10]^

### 2.2 Measures

Clinical data collection consisted of sending the questionnaire to all participants of the study, keeping track of the participants (i.e., using telephone registries), and sending a one-off reminder asking for the questionnaire according to the ethical approval. Collection of socio-demographic data included questions on working capacity (hours/week) before and after the accident, after returning home post rehabilitation, and during the further course. Comorbidities were recorded during interview and confirmed by medical records (Table 1).

**Table 1:**
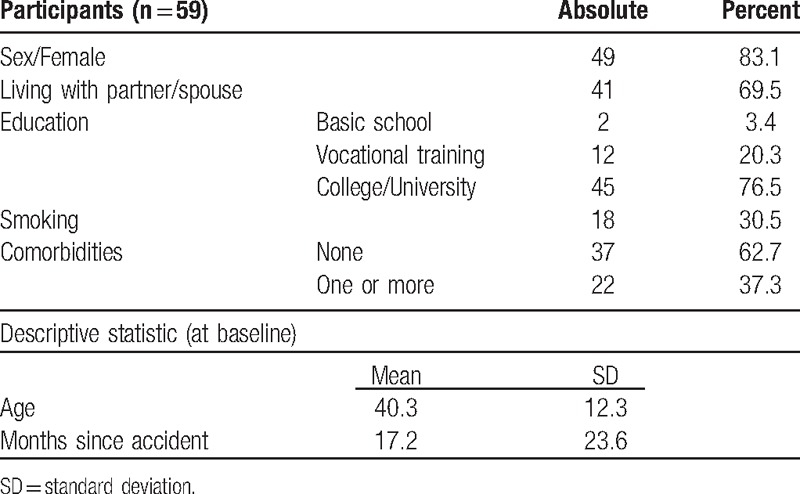
Sociodemographic data of participants at baseline/entrance.

Standardized outcome measurements consisted of different instruments. The German version of the Short Form 36 (SF-36) assessed health and quality of life in terms of physical, mental, and biopsychosocial health.^[14,15]^ The SF-36 is the most widely used health-related quality of life instrument, having been implemented in numerous studies in over 172 languages. Normative data were derived from a German population survey, thus allowing stratification by sex, age, and comorbidities (none vs. 1 or more).^[16]^ To inquire into cervical spine-specific limitations the North American Spine Society's (NASS) cervical spine self-assessment questionnaire was used (NASS pain, NASS function, NASS pain and function, and NASS neurogenic symptoms).^[17,18]^

Since the SF-36 proved more responsive in mood assessment than the HADS (hospital anxiety and depression scale), the HADS was removed from the original questionnaire set for the 60 months follow-up.^[19]^ From the coping strategies questionnaire (CSQ) only “Catastrophizing” was used at the 60 months follow-up for the same reason. All scores (SF-36, NASS, CSQ) were scaled 0 to 100; 0 reflects worst and 100 best. For example, SF-36 bodily pain = 100 reflects no pain.

### 2.3 Analysis

Assessments were performed on entry and on discharge from the rehabilitation clinic as well as at 6 and 60 months after entry. Analyses were performed using the statistical software package SPSS 22 for Windows (SPSS Inc., Chicago, IL).

The effects were quantified by ES according to Kazis and standardized response mean (SRM) according to Liang et al.^[20,21]^ The score difference between follow-up and baseline divided by standard deviation of baseline is defined as ES according to Kazis et al.^[20]^ The score difference (follow-up − baseline) divided by standard deviation of the score differences (follow-up − baseline) is defined as the SRM.^[21]^ An ES/SRM >0.80 is considered large, 0.50 to 0.79 moderate, 0.20 to 0.49 small, and 0.00 to 0.19 very small. A positive ES/SRM reflects improvement. Significance tests were performed using the non-parametric Wilcoxon–Mann–Whitney *U* test on the score differences baseline to follow-up (pervise intraindividual differences). At 5 years, the SF-36 scores were compared with the norms (first aim). Between 6 and 60 months, score changes were quantified by ES and SRM and the scores at 6 and 60 months were compared with the Wilcoxon–Mann–Whitney *U* test (second aim).

To assess possible selection bias, data between entry and the 6 months follow-up of the completers were compared with data of those who dropped out after the 6 months follow-up.

## 3 Results

**Figure 2:**
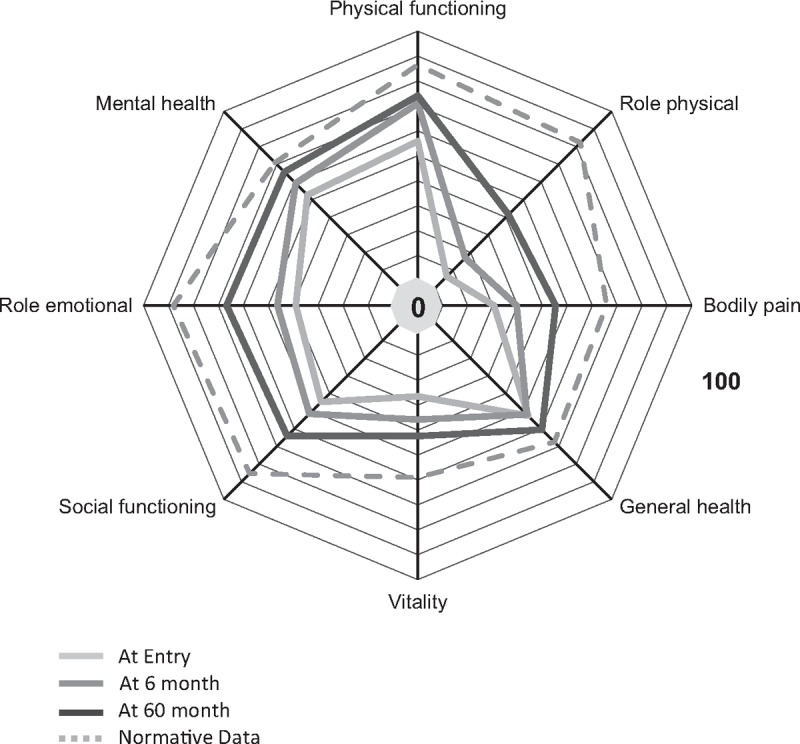
Results Short Form 36. 0 = worst, 100 = best.

**Figure 3:**
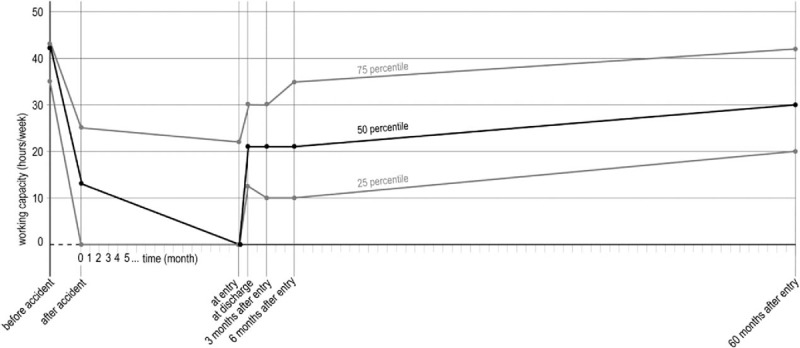
Working capacity (hours/week).

Outcome measures are shown in Table 2. Out of 115 initial participants, 59 (51%) complete data sets could be derived for the 60 months follow-up study (Fig. 1). The baseline sociodemographic data of the participants (at the 60 months follow-up) are shown in Table 1. The median patient has no comorbidities, is woman, well educated and living in a partnership. The average age was 40.3 years. Time since the accident averaged 17.2 months. Results are shown in Table 2 and Fig. 2, working capacity in Fig. 3.

**Table 2:**
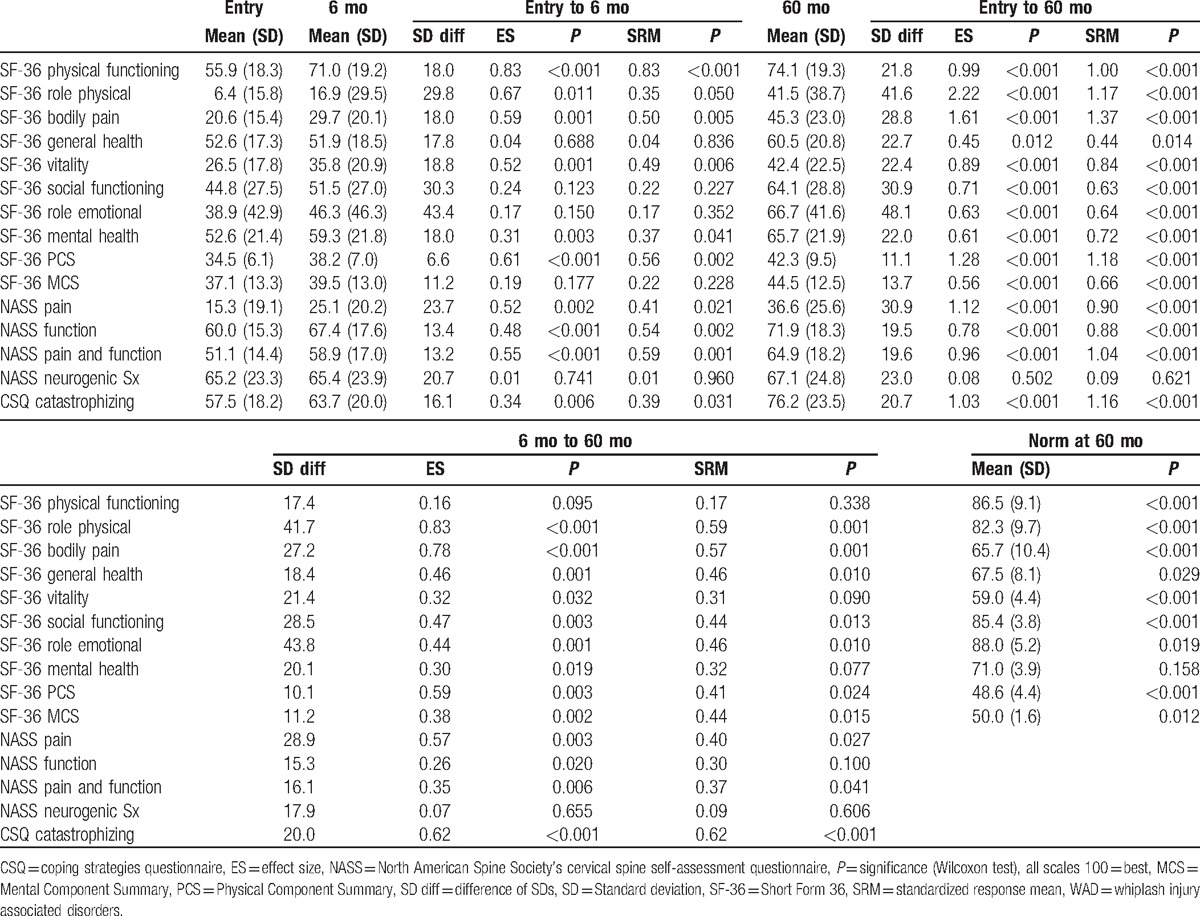
Long-term course of WAD patients (n = 59) after inpatient rehabilitation.

Comparing data between entry and 60 months after rehabilitation revealed that the ES/SRM of 8 of 15 parameters were large. Bodily pain improved on the SF-36 questionnaire from mean 20.6 at entrance to 45.3 (100 = no pain) at 60 months with ES = 1.61 and SRM = 1.37. ES = 1.12 and SRM = 0.90 within the NASS pain scale (mean at entry 15.3, at 60 months 36.6) also reflects a large improvement. Catastrophizing in the CSQ changed from 57.5 at entry to 76.2 at 60 months after rehabilitation with ES = 1.03 and SRM = 1.16. Physical functioning and role physical of the SF-36 as well as function within the NASS showed large positive ES/SRM values. Significance was very high (*P* < 0.001) for all of the above listed scales. Neurogenic symptoms did not change over the course.

The median working capacity increased from 13 h/wk after the accident to 30 h/wk after 60 months.

Outcome between 6 and 60 months after rehabilitation showed small to moderate ES/SRM values. SF-36 bodily pain (mean 29.7 at 6 months) improved with ES = 0.78 and SRM = 0.57. NASS pain score (mean 25.1 at 6 months) changed with ES = 0.57 and SRM = 0.40. CSQ catastrophizing (mean 63.7 at 6 months) advanced with an ES/SRM = 0.62. SF-36 role physical (mean 16.9 at 6 months) had a large ES = 0.83 and moderate a SRM = 0.59. NASS function (mean 67.4) had a small ES = 0.26 and a SRM = 0.30. SF-36 physical functioning (ES = 0.16) and NASS function (ES = 0.26) showed only small improvements. The same is true for SF-36 vitality (ES = 0.32).

Nine of 10 scales on the SF-36 remained below the level of the normative German population. Role physical showed the largest difference of the means (40.8) followed by social functioning and role emotional (both 21.3). Mental health was the only scale that did not differ from the norm (5.3, not significant).

The median working capacity remained constant at 21 h/wk within the period after discharge until the 6 months follow-up. It increased from 9 h/wk at 6 months to 30 h/wk at the 60 months follow-up.

Sensitivity analysis showed that more women participants finished the study (odds ratio 1.79, *P* = 0.20). An average of plus 3.3 years age for the completers (n = 59) of the study (participating in the 60 months follow-up) compared with those who dropped out at 6 months (n = 56) was observed. The time since the accident of the completers was on average 4.5 months longer than that of the non-completers. However, neither of the differences attained statistical significance (*P* = 0.129 and *P* = 0.181). The same was true for all baseline scores and score changes of the SF-36 and NASS comparing completers versus non-completers between baseline and 6 months. For example, the mean baseline score of the completers was 20.6 SF-36-bodily pain versus non-completers 21.4 (*P* = 0.755). At 6 months, the score changed by a mean of 9.1 for completers versus 9.0 for non-completers (*P* = 0.979) on the same scale.

## 4 Discussion

After having shown moderate to large improvements up to 6 months after entry to rehabilitation, the main aim of this study was to examine the further course in the long-term.^[10]^ Between 6 and 60 months, further significant improvements especially on pain, pain-related catastrophizing, role physical and psychosocial scales were observed. Function improved only by small effect sizes and neurogenic symptoms did not change. For the sum of all assessment time points between entry and 60 months, large improvements resulted on most of the relevant scales and the median working capacity increased from zero at entry to up to 30 h/wk after rehabilitation. The working capacity showed a substantial increase between 6 and 60 months while it remained constant in the previous phase (at discharge from rehabilitation, 3 months and 6 months after rehabilitation).

There are few long-term studies covering a period of 5 years or more and reporting quantitative outcome data. A Danish study prospectively assessed whiplash patients immediately and 12 months after whiplash trauma.^[22]^ For each patient (case) 5 matched controls were selected in the general population. Various risk factors were then retrospectively evaluated up to 5 years prior to trauma and 15 months after. Experiencing a whiplash trauma was associated with future negative change in provisional situation at the 12 months follow-up (odds ratio 3.13). Receiving sickness benefits for more than 12 weeks during the 5 years preceding the collision was associated with considerable neck pain 1 year after the accident by odds ratio 3.34. A Swedish cross-sectional study followed up 158 patients 5 years after whiplash injury by validated questionnaires (i.e., the Becks depression inventory and the neck disability index [NDI]).^[23]^ The participants were grouped into recovered, mild, moderate/severe disability using the NDI. Multivariate, logistic regression analysis showed only one statistically significant association between mild to severe disability and recovered (NDI ≤ 8) and depression (odds ratio 1.26). However, no effect data are available by that study design. In 1998, a Swedish study with 40 patients post-whiplash trauma and 33 patients with musculoskeletal pain of the neck and back showed improvements with a small ES in coping (0.45) and life-satisfaction (0.40) 2 years after participating in a 6 weeks outpatient rehabilitation program for the whiplash group.^[6]^

While this study revealed improvements between the mid-term and the long-term follow-up, which are consistent to our findings, another study reported almost no changes between 1 and 6 to 8 years after whiplash on the NDI and an increase in kinesiophobia as measured by the Tampa scale.^[24]^ During the first year after trauma patients received outpatient physiotherapy (66%), massage (5%), and various analgetics (66%) and were instructed to “act as usual.” Structured multidisciplinary rehabilitation might have additional positive long-term effects compared with usual outpatient management. Beyond the natural course, a certain part of the improvements might be attributed to the rehabilitation. In a randomized controlled cohort study reflecting the natural course of the WAD, waiting list patients (n = 18) significantly worsened while neck specific exercise participants (n = 23) significantly improved between baseline and the 3 months follow-up.^[25]^ The period between accident and baseline was on average 22 months in that study and 23 months in our study (at the 6 months follow-up). Subsequent observed improvements may indicate an effect of rehabilitation when compared with the natural course. A Finnish study collected data of 10,412 traffic accidents with 508 reports of neck injury victims leading to 144 replies at the 3 years follow-up.^[26]^ Interventions were not specified. It was concluded that the proportion of people who experience significant health deterioration compared with state of health before the accident remains unchanged after 3 years and that WAD classification was significantly associated with poor outcome after 3 years. The return-to-work state of persons sick-listed due to WAD (n = 104) compared with those sick-listed due to other musculoskeletal disorders (n = 3204) was analyzed in a Dutch 3 years follow-up study.^[27]^ Of the WAD group, 34% returned after 1 year, 44% after 2 ,and 43% after 3 years (cumulative rates). An explanation from the authors for stagnation between 2 and 3 years was that granting disability pension in Denmark requires permanently low work ability.

In Switzerland, disease management of WAD is individually tailored, as is the possibility for compensation. This has been highlighted in a current judicial decision of the federal tribunal of Switzerland (3rd June 2015, II Sozialrechtliche Abteilung) concerning the eligibility of chronic pain patients and others for invalidity pension. This is making the long-term outcome of treatment of whiplash patients of interest to the government, insurance companies, and pension funds. Invalidity pension is consists of a full or partial financial compensation for not being able to work at full capacity.

The strengths of this study were the long observation period of 5 years, the use of standardized questionnaires that measure health in a valid, comprehensive, and specific manner. A further strength of the study was comparison of the outcome to normative data. Documentation of working capacity exceeded the whole course with assessment before and after the accident. This may be of special interest to public health authorities and health insurance companies.

A first limitation may be the relatively small number of included patients at baseline, although comparable studies have even smaller sample sizes. A second limitation was the high dropout rate at 48.7% of the 115 initial participants. Efforts to keep dropout rate low have been made (keeping track of the patients, sending out a onetime reminder for the questionnaire). Possible reasons (i.e., high disease burden, conflicts with health insurance companies) for the high dropout rate have been discussed in the initial study.^[10]^ The long course of this study probably is an additional reason for the high dropout rate. Sensitivity analysis revealed no significant differences between completers and non-completers between baseline and 6 months follow-up. However, it cannot be excluded that the outcome of the non-completers was different from that of the completers between 6- and 60 months follow-up, that is, whether drop out was associated with long-term outcome. The observational study design and the lack of a control group did allow quantification of the changes of health but did not allow the deduction of a causal relation to the effectiveness of the treatment. However, the large observed positive effects might be associated at least temporally to the rehabilitation to a certain amount and can hardly be explained by any undeterminable effects of the spontaneous course alone, but the effects attributable to the intervention cannot be exactly quantified. In this sense this study is a naturalistic long-term study. The interdisciplinary program included organization of subsequent management for the period immediately after hospital dismissal, especially home exercise and advice about physio- and psychotherapy, as described in the previous outcome study.^[10]^ However, quality and quantity of adherence to those advises as well as general levels of physical activity were not assessed. Therefore, a regression analysis including exercises and coping strategies could not be performed. In contrast, working capacity of participants decreased from 13 to 0 h/wk under outpatient treatment between accident and rehabilitation median.

## 5 Conclusion

After large improvements in health and working capacity from baseline to the mid-term (6 months), further moderate to large improvements were observed in the long-term course (between 6 and 60 months), especially in pain, catastrophizing, and physical role performance. Residual impairments and symptoms persisted compared with the norms at 60 months. It is worth investigation if part of the positive effects can be attributed to the interventions during inpatient rehabilitation, for example, mediated by improved coping strategies. Nevertheless, another part of the effects might be explained by the natural course of the disease. This could not be quantified due to the naturalistic, uncontrolled study design.

## Acknowledgments

The authors wish to thank Ms Joy Buchanan for helping to prepare the manuscript.
